# The Concept of Harm in Medical Ethics

**DOI:** 10.1111/bioe.70124

**Published:** 2026-06-24

**Authors:** Marie‐Christine Fritzsche, Charlotte Franziska Unruh

**Affiliations:** ^1^ Institute of History and Ethics in Medicine, TUM School of Medicine and Health Technical University of Munich Munich Germany; ^2^ Department of Science, Technology and Society (STS), School of Social Sciences and Technology Technical University of Munich Munich Germany; ^3^ Department of Philosophy University of Southampton Southampton Hampshire UK

**Keywords:** artificial intelligence, biomarkers, clinical ethics consultation, counterfactual account of harm, ethics, harm

## Abstract

The concept of harm is central to medical ethics. In particular, it is frequently used in ethical deliberation about clinical cases and harm‐benefit analyses. Despite its significance, there has been surprisingly little discussion in practice about conceptual approaches and the nature of harm in medical ethics analyses. We examine how different conceptual accounts of harm shape ethical decision making through examples from clinical ethics consultation and data‐intensive medical innovations. By applying three prevailing theoretical‐philosophical frameworks—the counterfactual, temporal, and non‐comparative accounts of harm—to these cases, we demonstrate that explicitly identifying and discussing stakeholders' varying concepts of harm is crucial for ethical analyses in medicine and healthcare. While harm is only one of many considerations influencing medical ethics decision‐making, our analysis reveals that different conceptualisations of harm can lead to substantively different practical outcomes. In this way, how we understand harm can affect recommendation processes, stakeholder communication, and assessments of quality of life and best interests. Without making different types of harm explicit, stakeholders might talk past each other when weighing harm against benefit, as what constitutes harm in one case may not be considered harm in another. We argue that explicit consideration of the nature of harm ensures comprehensive ethical deliberation, and integrates multiple perspectives in healthcare settings. Our findings suggest that the ethical importance of conceptual analysis of harm extends beyond mere theoretical interest, with direct implications for clinical practice and ethical decision making in healthcare.

## 1 Introduction

Harm is a concept that is frequently used in medical ethics. The Hippocratic oath asks doctors to abstain from harming patients [1]. Non‐maleficence is one of the four principles of biomedical ethics [2]. Decisions about health spending and public health are made considering the balance of harms and benefits that can be obtained by some measure or investment. Given the centrality of the concept of harm for medical ethics, there is, however, surprisingly little discussion about the approaches on the nature of harm unlike in normative philosophy where there have been longstanding debates about how we should understand ‘harm’ and, indeed, whether any of the proposed definitions of harm are fit for purpose. These debates could be fruitful for medical ethics and lead to interesting conclusions. To our knowledge, however, the accounts of harm that have been developed in normative ethics have not yet been applied to cases in medical ethics in the way we do in what follows, nor with this particular argumentation.

This article aims to fill this gap. In doing so, we make two main points. To begin with, we show that it matters how we understand harm in medical ethics. The definition of harm that is used can make a significant difference to the ethical evaluation of cases. Following this, we argue that the lack of clarity about the concept of harm across debates in medical ethics is problematic. In particular, it often remains unclear which circumstances are covered when norms and principles in medical ethics that refer to harm prevention and avoidance are applied in practice.

The contribution this article makes, then, follows from these points. Firstly, we draw attention to the concept of harm that is central to medical ethics yet remains frequently undertheorised. We illustrate how different views on the nature of harm can influence the assessment of cases in medical ethics. Secondly, we recommend that contributions to medical ethics analyses make their understanding of harm explicit, to ensure conceptual clarity, prevent misunderstandings, and enable arguments and theories to be tested on the basis of different accounts of harm.

Importantly, we do not defend any particular theory of harm in this article or recommend any theory of harm for use by medical ethicists. Instead, our aim is to stimulate further research and to offer guidance for medical ethicists and ethical consultants who wish to draw on conceptual resources offered by the literature on harm in normative ethics.

The article proceeds as follows. We begin by explaining the main accounts of harm in normative and applied ethics (Section 2). We then explore the role of harm in medical ethics, suggesting that the concept of harm is not merely part of principles guiding medical ethics but is also used in practical ethical reasoning and decision making in medical contexts (Section 3). We then bring these points together by illustrating, using different case scenarios, how different accounts and normative assumptions about the nature of harm can significantly influence theoretical consideration and evaluation. We conclude by formulating recommendations for further research and practice in medical ethics.

## 2 Theories of Harm

There are two questions about harm. First, what does it mean for someone to be harmed? That is, under which conditions does someone count as suffering harm? Second, what does it mean for someone to harm someone else? That is, under which conditions can someone be said to be harming someone else?^1^ The first question concerns *well‐being*: it asks us to define when an impact on a patient's well‐being counts as harm. The second question concerns *responsibility*: it asks us to define when an agent is responsible for harm. In this article, we focus on the first question.

In the philosophical literature on harm, three main accounts are commonly distinguished:

Counterfactual: A suffers harm if and only if A is worse off than A would otherwise have been (e.g., [3, 7, 8]).

Temporal: A suffers harm if and only if A is worse off than A was before (e.g., [9, 10]).

Non‐comparative: A suffers harm if and only if A is badly off (e.g., [5, 11, 12]).

The three views, as presented here, are incomplete. A full account of these views must fill in the details: what is the counterfactual scenario we are supposed to imagine? What is the earlier time we should use to compare A's present well‐being with? And when does someone count as being ‘badly off’? Depending on the answer to these questions, different variations of counterfactual, temporal, and non‐comparative accounts emerge. Nonetheless, the views as presented are sufficient for our aims here: to illustrate how different responses to the question ‘What is harm?’ can have significant implications for debates in medical ethics.

To understand the difference between the three views, consider:

Punch: Ana punches Bob, leading to painful swelling around the eye.

Clearly, Bob suffers harm. According to counterfactual accounts, Bob suffers harm because Bob is worse off than Bob would have been, had Ana not punched him. According to temporal accounts, Bob suffers harm because Bob is worse off than he was before Ana punched him. According to non‐comparative accounts, Bob suffers harm because Bob is in pain, and thereby badly off.

In this case, all three accounts provide the correct result. However, all three accounts face counterexamples. Consider:

Pre‐empted punch: Ana punches Bob, leading to painful swelling around the eye. Had Ana not punched Bob, Celia would have punched Bob with the same result.^2^

Birth condition: Ana administers medication to Bob before Bob is born, which leads Bob to experience pain.^3^

Loss of vision: Bob has excellent vision. Ana punches Bob, which causes Bob's vision to slightly worsen on one eye. Bob's vision remains well above average.^4^

In all three cases, intuitively, Bob suffers harm. However, the counterfactual account finds no harm in Pre‐empted punch, since Bob would have suffered harm even if Ana had not punched him. The temporal account finds no harm in Birth condition, since Bob was never better off than before he was born with the painful condition. The non‐comparative account finds no harm in Loss of vision, since Bob is not intrinsically badly off.

These problems do not amount to decisive objections. There are responses and amendments that could be made by proponents of these accounts.

In many real‐world cases, the three accounts of harm deliver the same verdict on cases. But as our examples illustrate, this will not always be the case. Moreover, different accounts of harm focus on different aspects of a situation that make it harmful, and so an agent might judge a situation differently, depending on the account of harm they employ. For example, while the temporal account and the non‐comparative account agree that hearing loss is harmful, they might disagree to what extent a patient is harmed by hearing loss, depending on the patient's previous hearing (temporal) and the hearing ability that is considered normal (non‐comparative). Therefore, it remains important to use the different concepts, explore which concept of harm is used in specific cases and by certain stakeholders and whether they lead to different outcomes. Finally, the philosophical debate contains well‐established objections against all accounts of harm (see [13] for an overview), suggesting that focusing on one account of harm over others will lead agents to misidentify harm in at least some cases. More recently, novel accounts of harm have been developed that claim to overcome problem cases (e.g., see [22] for a ‘hybrid’ account that combines a temporal and a non‐comparative element).

In sum, there are three main accounts of harm in the philosophical literature. While the accounts agree about paradigm cases of harm, they have different results in other cases. Which of these accounts, if any, is correct is a matter of live debate in the philosophy of harm, with no consensus in sight. This illustrates the difficulty of defining harm—a difficulty which, we contend, has implications for medical ethics.

## 3 Conceptualising Harm in Medical Ethics

In many analyses in medical ethics, for example in medical research, technology development for healthcare or clinical settings, harms are identified and analysed that occur or can occur in specific contexts. Here, ‘harm’ often remains undefined.

For example, the four‐principle‐model by Beauchamp and Childress [2] has dominated bioethics over the last few decades.^5^ One of the principles is non‐maleficence. When applying this principle, medical ethicists identify potential harms. However, the *concept* of harm itself is rarely defined or examined in these contexts.^6^ We claim that doing so can help us to identify harms that might otherwise have been overlooked and accommodate different ways in which the concept of ‘harm’ might be understood by patients, practitioners, and medical ethicists.^7^

We argue that reflection on the concept of harm, and clarification of the concept where it is used, would benefit analyses in medical ethics. A better understanding of what ‘doing no harm’ involves can also inform harm‐benefit analyses and the specifying and weighing of principles in applied contexts.^8^

One might object that it is sufficient if medical analyses rely on a common‐sense understanding of harm. However, we suggest that there are cases in which what counts as harm is not straightforward. In these cases, conceptual distinctions are needed.

In the following, we illustrate this point using two examples: one from clinical ethics consultation and one from medical research involving data‐ and AI‐driven biomarkers^9^. To repeat, the goal here is not to defend a particular account of harm, but rather to show that different concepts of harm can lead to drastically different implications in practice.

### 3.1 Clinical Ethics Consultation

We begin with an example from a clinical setting, specifically from clinical ethics consultation in intensive care. This is a typical scenario for clinical ethics consultations. Here, we refer to an example by Shea [27], which we have adapted.

“[A] decisionally incapable […] middle‐aged man […] [is] admitted to the hospital after a major stroke and is now receiving life‐sustaining treatment. He is expected to survive with continued aggressive treatment, but he has suffered cognitive and physical deficits and will be permanently impaired. The medical team has no information concerning the patient's wishes and no surrogate decision maker, and they must decide whether to continue life support based on whether it will be in the patient's best interest and what his future quality of life will be.” [11, p. 447]

In this case, it is not only important to discuss the meaning of ‘best interest’ or ‘quality of life’, but also the connected question of what ‘harm’ would mean here (see Shea [27]). Before analysing different conceptions of harm, we note that without consent to ‘do not resuscitate’ or any other clear statement to end the life support, it would typically be continued as standard of care. In the following, we abstract from this and present different accounts of harm that different people might still experience in this situation, depending on which concept of harm they employ.

Different accounts to harm can lead to fundamentally different ethical analyses of this case. To illustrate, imagine that different people involved in the patient's care differ in how they understand harm, and consequently, what they view as a burden and diminishment of quality of life for the patient.^10^

The leading neurologist of the team implicitly uses a counterfactual view of harm by comparing the patient's present condition with what would have been the case without intervention. On that view, the continuation of life‐sustaining measures does not represent harm, as the alternative (discontinuation) would lead to death. The central comparison is drawn between the patient's present condition vs. the counterfactual scenario in which treatment was discontinued.

The patient's partner uses a temporal approach to harm. She makes a comparison between the patient's current condition and their pre‐stroke condition and considers ongoing interventions as possibly harmful as they prolong a condition that is significantly worse than their previous condition. According to this view, the maintenance of life‐sustaining interventions could present harm as they prolong a period during which the patient's well‐being deteriorates compared to their previous state.

The clinical ethicist follows a non‐comparative view of harm, which centers on whether the patient's current condition represents an intrinsically harmful state, independent of comparisons with other scenarios. From this perspective, the severe physical and cognitive impairments could constitute harm, regardless of comparisons with previous conditions or alternative scenarios.

This case shows why it is important to make conceptual assumptions about harm explicit although clinical ethics consultation is and must be very practice oriented, as there is often limited time to make critical ethical decisions. Stakeholders (typically a clinical ethicist and healthcare professionals, sometimes also patients/next of kin) often have different views and cultural backgrounds, and might also have different concepts of harm.

In the process of analysing an ethical case with the four‐principle model in the context of a clinical ethics consultation [31], the individual weighs each principle carefully using specific arguments related to the case to determine their relative importance and relevance compared to the other principles. Note that it is not common to make one's concept of harm explicit before starting the ethical analyses in clinical settings. This is true for the application of the principle of non‐maleficence (doing no harm) and also for overarching analyses of harms and benefits. In this context, we argue, an explicit consideration of different accounts of harm has at least two advantages.

First, including patients' understanding of harm in ethics analyses could ensure that the will of patients is better incorporated and that misunderstandings are avoided. While the role of patients varies in different settings of clinical ethics consultation services [32], in fact, patient participation is not a standard in clinical ethics support services in Europe [33]. Other considerations aside, for designing a good participatory approach for clinical ethics consultation [34], incorporating patients' perspectives on concepts used in the consultation process can make a crucial difference to the outcome. As our example illustrates, a more participatory approach would need to also include the patients' perspectives on harm in clinical ethics consultations, which can take place before and/or during the main discussions of the clinical ethics consultation.^11^

Second, reflecting on and including different understandings of harm in clinical ethics analyses can mitigate the risk of overlooking harms. Overlooking harms might mean that certain needs are neglected or that ethical issues are not identified or addressed. Moreover, it might lead to inaccuracies in harm‐benefit analyses. After all, if some harms are not recognised as such, it can seem as if certain benefits outweigh them—which in turn might lead ethicists to underestimate risks of harm. The opposite may also apply: if we do not have a clear notion of *benefits*, then we might overlook some benefits, which can lead to a similarly unbalanced analysis. Given that decisions in high‐risk fields such as medicine and healthcare often have far‐reaching implications for the stakeholders involved, it is important to involve stakeholders, and (as we have argued) to consider their understanding of harm in doing so.

### 3.2 Research and Development in Healthcare

Being explicit about the concept of harm is not only relevant in clinical ethics consultation. To illustrate, here is an example from research and development of data and AI‐driven biomarkers in medicine where the first author has been a member of the ethics research group.

In an international research consortium data‐/AI‐driven tools are developed for precision medicine, more specifically biomarkers for early prediction and monitoring of a chronic inflammatory skin disease using machine learning and multi‐omics data as well as clinical data.^12^ To address ethical issues and mitigate potential harm early on, ethical analyses are conducted throughout the research and development process of data‐driven biomarkers in medicine (e.g., following an embedded ethics approach [38, 39, 40]).

Hypothetically, during the development of biomarkers, three stakeholders could use different concepts of harm:

A patient representative considers the impairment of human flourishing itself as harmful. She explains that she experienced severe symptoms in her childhood that hindered her from pursuing her desired career. When evaluating the potential harms and benefits of data‐driven tools that could enable better disease prediction and earlier treatment, she considers not only risks such as potential biases that could perpetuate inequalities, but also the impediment of flourishing throughout her life, which might be mitigated with more targeted treatments. She employs a non‐comparative account of harm.

The researchers, data‐scientists and AI‐developers identify harm in cases where data‐driven tools create adverse effects that would not have existed without them. Although the improvement of chronic disease using more targeted treatments would be classified as a benefit, under this counterfactual comparative concept of harm, the impairment of human flourishing through the chronic disease might not be classified as harm and could be overlooked in harm‐benefit analyses when assessing the tool. Consequently, and maybe paradoxically, without including the patient representative's perspective in the ethical evaluation, the tool might be assessed more negatively than with her contribution.

The clinical researcher uses a temporal view of the harm. She compares the condition of patients before and after applying the tools, focusing on disease progression rates compared to baseline, changes in quality of life over time, treatment effects compared to previous treatment approaches, and long‐term effects on patient well‐being.

This example shows that being more explicit about underlying conceptions of harm could help to make analyses in medical ethics more generally (not only in clinical ethics consultation) more precise. It could enhance the identification of harms that are weighed against benefits and evaluated alongside other ethical dimensions, thereby leading to more precise analyses. This example also reinforces the point, already apparent above, that involving a plurality of views, including the views of those affected, fostering participative approaches, and more transparency about the concept of harm that is used by researchers, clinicians, and other stakeholders might also uncover ‘hidden’ harms, that is, valid perspectives on harm that might not be obvious to all stakeholder groups. In our example, a patients' comments on the harm they experience or anticipate might not be understood by other members of the research team, since they use the term ‘harm’ to refer to different concepts.

Overall, we have shown that scenarios can be evaluated differently depending on which concept of harm is applied. As we have seen, some cases are classified as harmful on one account of harm, but not on others. We argued that a more nuanced understanding of harm in medical ethics could improve the identification of harms that might otherwise remain invisible to certain stakeholders. This is the cornerstone to addressing these harms and mitigating them in a more targeted way. Different conceptions of harm in decision‐making can reveal moral disagreements. If harm is left undefined—such that what one understands by it is not made explicit—it can happen that this moral disagreement/what exactly one considers good and bad, right and wrong, not clearly come to light.

To sum up, we submit that our discussion gives rise to three conclusions:

1. Different conceptions of harm may lead to different recommendations and different decisions: This is clearly illustrated with our first example. In this case, we can imagine that the neurologist might recommend continuing the treatment because, from her counterfactual perspective, it does not constitute harm. The patient's partner could advocate discontinuation of treatment based on the temporal comparison with the patient's previous condition. Finally, the ethicist could emphasise the absolute degree of impairment that affects the patient and recommend a different course of action.
2. The assessment of quality of life: Different concepts of harm can lead to different assessments of quality of life and best interests. This is because whether, and to what extent, a state of affairs harms a patient affects what quality of life that state of affairs offers the patient, and whether putting the patient in this state is in the patient's best interest. To illustrate this point by drawing on our first example, a counterfactual approach might focus on preventing death to avoid the counterfactually worst outcome for the patient. In contrast, a temporal approach might focus on avoiding prolonged states of impaired functioning. These priorities might conflict in cases where, for example, available treatment options differ in how likely they are to prevent death, and how likely they are to maintain or restore capabilities.
3. Integration of multiple perspectives: The first example shows how a hybrid account of harm (as suggested by Unruh) can be beneficial in clinical ethics consultation by considering both non‐comparative and comparative aspects of the patient's situation.^13^ By explicitly ensuring that different accounts are considered, ethics consultants moderating a discussion, for example, could uncover differences in how harm is understood by stakeholders, facilitate a more nuanced ethical analysis of the situation, and ensure that recommendations reflect accurately the perspectives of stakeholders.

## 4 Conclusion

Harm is, of course, only one of many considerations that influence decision‐making and analyses in medical ethics. However, it is an important consideration. We have argued that making our concept of harm explicit is crucial for ethical analyses in medicine and healthcare. The ethical importance of the conceptual analysis of harm extends beyond mere theoretical interest, as our examples demonstrate. Different concepts of harm lead to substantively different practical outcomes. Our analysis demonstrates that the application of a particular concept of harm can have concrete implications for clinical practice and research. (Note that, although we do not have the space to discuss this here, we submit that our arguments apply in an analogous way to the concept of benefit.)

Rather than seeking a single ‘right’ concept of harm, we suggest that future investigations could examine how different contexts may require different concepts of harm, and how multiple concepts can productively coexist and be combined. The key is making explicit and transparent which concepts of harm are used and what follows from specific concepts of harm. By using examples from clinical ethics consultation and data/AI‐driven tools, we have shown the importance of developing a more nuanced understanding of harm for use in ethical analyses in medicine and healthcare. This promises to deliver significant practical benefits, such as enabling more comprehensive ethical analyses and including more perspectives in decision‐making, enabling more precise harm‐benefit analyses and better informed decision‐making.

In sum, we contend that ethical analyses in medicine and healthcare would benefit from more systematic questioning of the assumptions behind key concepts such as harm. This becomes increasingly important as new technologies and practices in healthcare continue to challenge traditional understandings of harm and benefit. Future work should explore how different frameworks for understanding harm can be integrated into practical ethical decision‐making and combined with existing models, while remaining sensitive to the demands of specific contexts such as time constraints in healthcare settings.

## Author Contributions

M.‐C.F. created the initial outline and wrote the great majority of the original article draft, subsequent drafts and the final manuscript. M.‐C.F. led the conceptualisation of the article and conducted the majority of analyses. C.F.U. contributed to conceptualising the article and contributed to writing, reviewing and revising the article drafts and the final manuscript.

## Conflicts of Interest

The authors declare no conflicts of interest.

## Data Availability

The authors have nothing to report.

## Endnotes

1 Harm and harming are distinguished, for example, by [3, p. 31], [4, p. 853], [5, p. 360], and [6, p. 890].

2 For similar cases, see [13, p. 398]. For defenses of counterfactual accounts, see [7, p. 295‐296], [14, p. 62], [15, p. 371], [16, p. 9–18], and [17, p. 14–15] (but see [18] for objections to such defenses).

3 Cases in which agents are harmed before their birth are discussed by [19, p.369], [20, p. 445–446], and [5, p. 370].

4 Cases in which a deterioration is slowed down are discussed by [19, p. 368], [21, p. 432].

5 Harm is also relevant in approaches other than the four‐principle‐model. For example, reflective equilibrium [23].

6 There is extensive literature in bioethics and philosophy on related concepts, such as suffering [24, 25] and well‐being [26]. We suggest that the connection between these and harm deserves further exploration.

7 The point that stakeholders might understand concepts in different ways is not new. Beauchamp and Childress [2] acknowledge that stakeholders might understand well‐being differently (without defining it themselves [27]).

8 The difficulty of weighing, balancing, and specifying principles has been discussed extensively (e.g., see 27, 28, 29]).

9 The FDA‐NIH Biomarker Working Group characterises a biomarker as “[a] defined characteristic that is measured as an indicator of normal biological processes, pathogenic processes, or biological responses to an exposure or intervention, including therapeutic interventions. Biomarkers may include molecular, histologic, radiographic, or physiologic characteristics. A biomarker is not a measure of how an individual feels, functions, or survives.” [30]

10 To avoid stereotyping, we would like to emphasise that these three views are not necessarily tied to these stakeholders.

11 If patients cannot communicate their will, and advance directives are not available, healthcare professionals frequently conduct harm‐benefit analyses. Here, it is relevant how harm is understood.

12 This example is based on recent ethics research in biomarker research and development in dermatology in the research consortium, where various ethical challenges were identified [35, 36, 37].

13 On Unruh's hybrid account, patients suffer harm when they are harmed in the non‐comparative sense and/or the temporal comparative sense.
